# MicroRNA Let-7a Inhibits Proliferation of Human Prostate Cancer Cells *In Vitro* and *In Vivo* by Targeting E2F2 and CCND2

**DOI:** 10.1371/journal.pone.0010147

**Published:** 2010-04-14

**Authors:** Qingchuan Dong, Ping Meng, Tao Wang, Weiwei Qin, Weijun Qin, Fuli Wang, Jianlin Yuan, Zhinan Chen, Angang Yang, He Wang

**Affiliations:** 1 Department of Urology, Xijing Hospital, Fourth Military Medical University, Xi'an, China; 2 Department of Biochemistry, Fourth Military Medical University, Xi'an, China; 3 Cell Engineering Research Center, State Key Laboratory of Cancer Biology, Fourth Military Medical University, Xi'an, China; Health Canada, Canada

## Abstract

**Background:**

Previous work has shown reduced expression levels of let-7 in lung tumors. But little is known about the expression or mechanisms of let-7a in prostate cancer. In this study, we used in vitro and in vivo approaches to investigate whether E2F2 and CCND2 are direct targets of let-7a, and if let-7a acts as a tumor suppressor in prostate cancer by down-regulating E2F2 and CCND2.

**Methodology/Principal:**

Findings Real-time RT-PCR demonstrated that decreased levels of let-7a are present in resected prostate cancer samples and prostate cancer cell lines. Cellular proliferation was inhibited in PC3 cells and LNCaP cells after transfection with let-7a. Cell cycle analysis showed that let-7a induced cell cycle arrest at the G1/S phase. A dual-luciferase reporter assay demonstrated that the 3′UTR of E2F2 and CCND2 were directly bound to let-7a and western blotting analysis further indicated that let-7a down-regulated the expression of E2F2 and CCND2. Our xenograft models of prostate cancer confirmed the capability of let-7a to inhibit prostate tumor development in vivo.

**Conclusions/Significance:**

These findings help to unravel the anti-proliferative mechanisms of let-7a in prostate cancer. Let-7a may also be novel therapeutic candidate for prostate cancer given its ability to induce cell-cycle arrest and inhibit cell growth, especially in hormone-refractory prostate cancer.

## Introduction

MicroRNAs (miRNAs) are endogenous, noncoding small RNAs 20–25 nucleotides in length [1], which play an important regulatory role through complimentary binding of the 3′ untranslated regions (UTRs) of target genes. Binding results in the degradation of the target mRNA and inhibition of translation [2]. Many miRNAs are associated with cancer, and are involved in cell development, differentiation, proliferation, and cell death [3]. Many reports have indicated that miRNAs can be useful for cancer diagnosis and therapy [4]. let-7 was first identified in *Caenorhabditis elegans* [5]. It is nearly undetectable in the embryonic stage of development, but becomes more abundant in later stages of development [6]. Previous work has shown reduced expression levels of let-7 in lung tumors compared to normal lung tissue. let-7 slows cellular proliferation by down-regulating the oncogenes RAS/c-MYC and HMGA-2 at the translational level [7], [8]. The same tumor suppressive functions have also been reported for let-7 in colon cancer [9].

*In silico* analyses of potential let-7a targets (www.targetscan.org andwww.microrna.org) reveal that both E2F2 and CCND2 are possible targets of let-7a. E2F2 and CCND2 are cell-cycle regulators and aberrant expression of them can lead to abnormal cellular proliferation. Our preliminary experiments indicate that protein levels of both E2F2 and CCND2 are up-regulated in the PC3 prostate cancer cell line. Little is known about the expression or mechanisms of let-7a in prostate cancer. In this study, we used *in vitro* and *in vivo* approaches to investigate whether E2F2 and CCND2 are direct targets of let-7a, and if let-7a acts as a tumor suppressor in prostate cancer by down-regulating E2F2 and CCND2.

## Materials and Methods

### Ethics Statement

All samples were obtained from patients who signed informed consent approving the use of their tissues for research purposes after operation. The clinic pathological factors of the 26 patient were showed in Table S1. The use of human tissues in this study was approved by the Institutional Review Board of the Fourth Military Medical University and was in accordance with their guidelines(No 2008039085). All experiments involving animals were conducted according to the Animal Welfare Act and approved by Animal Care and Use Committee of the Fourth Military Medical University. (Approval number 200804052353).

### Cell culture and tissue collection

Human prostate cancer cell lines LNCap, DU145, PC3, and PrEC (prostate epithelial cells) and human embryonic kidney cells HEK293A were obtained from American Type Culture Collection (ATCC, Manassas, VA, USA). Cells were cultured in RPMI-1640 (Gibco) supplemented with 10% fetal-calf-serum and penicillin (100 U/ml). Cultures were maintained under an atmosphere containing 5% CO_2_ (Forma Scientific). Twenty-six freshly resected prostate cancer specimens and their adjacent non-tumorous specimens were collected from the Department of Urology in Xi'jing Hospital. The specimens were immediately frozen in liquid nitrogen and maintained there until use.

### Plasmid construction and cell transfection

Let-7a was amplified and purified by miRNA isolation kit (Invitrogen, Carlsbad, CA) according to manufacturer's protocol. PCR primers for let-7a were: *5′-GAATTCTCACACAGGAAACCAGGA-3′* (forward) and *5′-CTGCAGTCAGGCATTTAAGTGACCG-3′* (reverse). Let-7a PCR products were cloned into the *Eco*RI/*Pst*I cloning site of a pcDNA3 plasmid containing green fluorescent protein (GFP) to form the plasmid pcDNA3-let-7a-GFP. Cell transfection was performed with lipofectamine 2000 (Invitrogen, Carlsbad, CA) according to the manufacturer's protocol. Briefly, PC3 cells were plated in six well plates and grown without antibiotics to 70–90% confluence. Each well received 6 µl of lipofectamine reagent containing 3 µg of plasmid DNA. Three wells received pcDNA3-let-7a-GFP plasmid while the other wells received the empty vector, pcDNA3-GFP. G418 (400 µg/ml) was added to cells 24 h after transfection. Mixed clones were screened and cultured for an additional 4 weeks. PC3 cells transfected with let-7a were designated as PC3-let-7a-GFP, the negative control (cells transfected with the empty vector) was designated as PC3-GFP. PC3 cells were also transfected with 30 nM of synthetic let-7a (mimics), and synthetic negative control miRNAs (NC). Lastly, a synthetic let-7a-inhibitor sequence and synthetic let-7a-inhibitor negative control (NC inhibitor). Sequences of synthetic hsa-let-7a, negative control, hsa-let-7a inhibitor and inhibitor negative control were showed in Text S1.

### Total RNA extraction and real-time RT-PCR

For let-7a, Total RNA was extracted from samples using a miRNeasy Mini Kit (Qiagen). cDNA was synthesized using TaqMan MicroRNA Reverse Transcription Kit (Applied Biosystems). Real-time RT-PCR was performed with TaqMan MicroRNA Assay kit (Applied Biosystems). The primer for let-7a was *5′-GAGGTAGTAGGTTGTATA-3′.* For E2F2 and CCND2, total RNA extraction and real-time RT-PCR were performed using SYBR® GreenER™ Two-step kit (Invitrogen, Carlsbad, CA). PCR primers for E2F2 were: *5′-GAGCTCACTCAGACCCCAAG-3′* (forward) and *5′-AACAGGCTGAAGCCAAAAGA-3′* (reverse). PCR primers for CCND2 were: *5′-AAGAATTCCTCCTCAATAGCCTGCAGCAGTA-3′* (forward) and *5′-GCGGGATATCGACCTGTGAGAATTCGAT-3′* (reverse). All protocols were carried out according to manufacturer protocols. The expression level of let7a was normalized to RNU6B. The expression level of E2F2 and CCND2 were normalized to GAPDH. Real-time RT-PCR was performed by using 7500 Real-time RT-PCR System (Applied Biosystems). PCR was performed under the following conditions: 50°C for 2 min, 95°C for 10 min, followed by 50 cycles at 95°C for 15 s, and 60°C for 1 min. Each sample was run in triplicate.

### MTT Assay

PC3 cells and LNCaP cells transfected with either NC or let-7a (mimics), or NC inhibitor and let-7a inhibitor were plated on 96-well plates at 1×10^4^ cells/well. Viable cells were measured 1, 2, 3, 4, and 5 days after plating. After incubation with 3-(4, 5-dimethylthiazolyl-2)-2, 5-diphenyltetrazolium bromide (MTT), the cells were lysed in 150 µl of 100% dimethylsulfoxide (DMSO) and UV-visible absorbance was read at 490 nm using the 96-well plate reader. Each sample was run in triplicate.

### Soft agar assay

Briefly, 2 ml of 0.5% agar was added to each well of a 12-well plate. Detached PC3-let-7a-GFP and PC3-GFP cells were mixed with the topagarose suspension (final concentration 0.3%), and then the cells were layered onto the 0.5% agarose underlay. The number of foci >100 µm were counted after 18 days. Each experiment was performed in triplicate.

### Cell cycle analysis

PC3 cells and LNCaP cells transfected with either NC or let-7a (mimics), or NC inhibitor or let-7a inhibitor were harvested 72 h after transfection, washed with cold phosphate buffered saline (PBS), and fixed in 1ml of 70% ethanol. After overnight incubation at 4°C in ethanol, cells were washed in PBS and suspended in 500 µl propidine iodide (PI) 30 min before flow cytometry. Populations in G0-G1, S, and G2-M phase were measured by flow cytometry (EPICS XL, Coulter, Miami FL) and the data were analyzed by using Multicycle-DNA Cell Cycle Analyzed Software. The measurement was performed in triplicate.

### Dual-luciferase reporter assay

To investigate whether E2F2 and CCND2 expression were regulated by let-7a, a dual-luciferase reporter assay was performed. The 3′-UTR of CCND2 and E2F2 were amplified by PCR respectively. Amplification of CCND2 used the following primers: *5′-GAATTCATGAGTTCTTCGTACTGG-3′* (forward) and *5′-GATATCCCATCATGAT GAGGATAC-3′* (reverse). PCR products were 398 bp and contained two binding sites for let-7a. The mutated 3′-UTR of CCND2 was synthesized after point mutations were made in several bases within the binding sites of these PCR products. Amplification of the 3′-UTR of E2F2 used the following primer sequences: *5′-GAATTCCTCTTCGACTCCTACGAC-3′* (forward) and 5*′-GATATCTGTGCTTCTT GGTACGTCG-3′* (reverse). The PCR products were 415 bp and contained two binding sites for let-7a. Mutated 3′-UTR of E2F2 were synthesized after several point mutations were made in the binding sites PCR products. After restriction digestion, amplified genes were cloned into the corresponding sites of a reconstructed pGL3 expression vector. The pRL-TK control vector expressing Renilla luciferase (Promega) was used for normalization of cell number and transfection efficiency. Cotransfections of synthetic let-7a sequence (mimics) and NC, or let-7a inhibitor and NC inhibitor were also performed in human embryonic kidney cells HEK293A by using lipofectamine 2000 (Invitrogen). Firefly and Renilla luciferase activity was determined by Pikkagene Dual Luciferase Assay System (Toyo-B-Net).

### Western blotting analysis

The following antibodies were used for western blotting: mouse anti-E2F2 (1∶200 dilution, Santa Cruz Biotech); mouse anti-cdk4 and mouse anti-cyclin D2 (1∶300 dilution; BD Biosciences, San Jose, CA); mouse anti-k-RAS (1∶300 dilution; Cell Signaling Technology, Beverly, MA); rabbit anti-β-actin (1∶4000 dilution, Sigma). Twenty-five micrograms of prostate cancer specimens and their adjacent non-tumorous specimens' protein as well as PC3-let-7a-GFP and PC3-GFP protein were electrophoresed in 10% SDS–PAGE minigels and transferred onto Hybond C nitrocellulose membranes (Amersham Life Science, Buckinghamshire, UK). After incubating with primary antibodies at 4°C overnight, the nitrocellulose membranes were washed three times with Tris-Buffered Saline Tween-20 (TBST) and then incubated with fluorecein isothiocyante (FITC)-conjugated, goat anti-mouse or goat anti-rabbit IgG (1∶500 dilution, Sigma) for 1 h at room temperature. After washing with TBST, blots were visualized by using the Odyssey Infrared Imaging system (LI-COR Bioscience, Lincoln, Nebraska USA). Each assay was repeated three times.

### Tumorigenicity

Five nude mice were subcutaneously injected with ∼1×10^6^ PC3-let-7a-GFP or PC3-GFP cells at a single site of the back. The weight of the tumor and the weight of the mouse were measured at the time of sacrifice (4 weeks after injection). Western blotting was performed to investigate whether E2F2 and CCND2 were down-regulated in nude-mouse xenograft model. Tumor suspensions were treated with lysis buffer and western blotting was done according to the procedures described above.

### Statistical analysis

Differences between each group were determined by the T test or Mann-Whitney *U* test using Statistical SPSS software package (SPSS Inc, Chicago) (*p*<0.05 was regarded as significant).

## Results

### Let-7a is down regulated in resected prostate cancer samples and in prostate cancer cells

**Figure 1:**
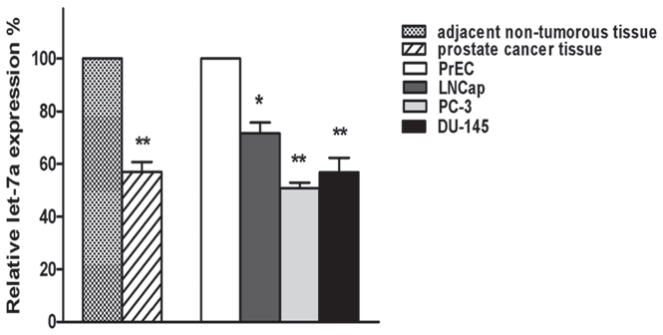
Let-7a expression in prostate cancer tissues and cells measured by real-time RT-PCR. Real-time RT-PCR show that the relative expression of let-7a in twenty-six prostate cancer samples, and in prostate cancer cell lines LNCap, PC3, DU-145 is higher than that in their adjacent non-cancerous specimens and the normal prostate epithelial cell PrEC. Data are shown as percent of mean signals in tissues and cells from three independent measurements. RNU6B was measured in parallel and used to normalize the expression level of let-7a in each experiment. Values represent means and error bars represent the SEM. (* *p<*0.01, ** *p<*0.01; Mann-Whitney-U test).

Real-time RT-PCR revealed that the expression levels of let-7a were decreased by ∼43% in resected human prostate cancer samples compared to the adjacent non-cancerous samples. The prostate cancer cell lines LNCap expressed only ∼70% as let-7a than normal prostate epithelial cells PrEC. Additionally, the amount of let-7a expression in PC3 cells and DU-145 were about 50% of that observed in PrEC. These results indicate that let-7a is decreased in prostate cancer, including the androgen-independent cancers PC3 (Fig. 1, * *p*<0.05; ** *p*<0.01).

### Let-7a inhibits cell growth *in vitro* and induces cell cycle arrest at the G0-G1 phase

**Figure 2:**
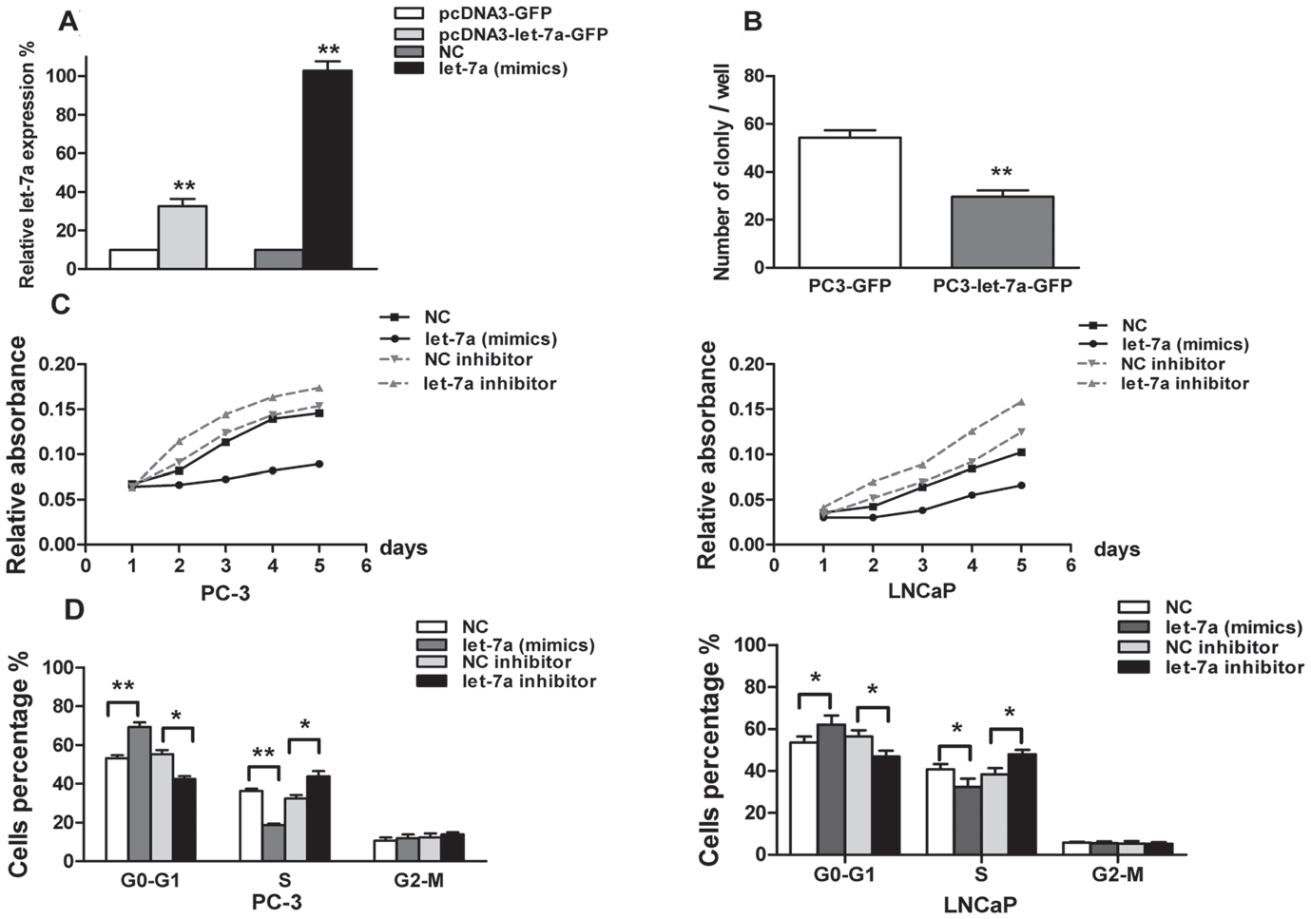
Effect of let-7a on growth and proliferation of PC3 and LNCaP. (**A**) Relative expression of let-7a in PC3 cells transfected with pcDNA3-GFP, pcDNA3-let-7a-GFP, NC, and let-7a (mimics). RNU6B was measured in parallel and used to normalize the expression level of let-7a in each experiment. Values represent means from three separate experiments and error bars represent the SEM. (** *p*<0.01; Mann-Whitney-*U* test). (**B**) PC3-let-7a-GFP and PC3-GFP cells were cultured in medium containing soft agar and incubated for 18 d. The number of foci >100 µm were counted. Values represent means from three separate experiments and error bars represent the SEM. (* *p*<0.01; Mann-Whitney-*U* test). (**C**) Viability of PC3 cells and LNCaP cells, which transfected with let-7a, NC or let-7a inhibitor, NC inhibitor was measured by MTT assays respectively. UV-visible absorbance was measured at 490 nm. Values represent means from three separate experiments and error bars represent the SEM. (* *p*<0.05; Paired-sample T test). (**D**) Analysis of cell cycle in PC3 cells after transfected with NC and let-7a (mimics) or NC inhibitor and let-7a inhibitor, as well as analysis of cell cycle in LNCaP cells after transfected with NC and let-7a (mimics) or NC inhibitor and let-7a inhibitor. Values represent means from three separate experiments and error bars represent the SEM. (* *p*<0.05, ** *p*<0.01; Mann-Whitney-*U* test).

After transfection, the expression level of let-7a in PC3-let-7a-GFP was ∼300% higher than in PC3-GFP by real-time RT-PCR; A ten-fold increase in expression of let-7a was observed in PC3 cells transfected with let-7a (mimics) versus those transfected with NC (Fig. 2A, *p*<0.01). This indicated that transfection was appropriately performed. Colony formation analysis indicated that the colonies formation ability of PC3-let-7a-GFP cells is ∼40% less than that of control cells (Fig. 2B, *p<*0.01). MTT growth curves indicated that from the second day, the survival of let-7a transfected PC3 cells and LNCaP cells are significantly less than that of negative control, and the survival of let-7a inhibitor transfected PC3 cells and LNCaP cells are significantly more than that of NC inhibitor transfected cells (Fig. 2C, *p<*0.05). Flow cytometry showed that the percentage of let-7a transfected PC3 cells and LNCaP cells in the G0-G1 phase was ∼30% (PC3) and 16% (LNCaP) higher than that of negative control, which paralleled with a ∼50% (PC3) and 20% (LNCaP) decrease in the S phase. the percentage of let-7a inhibitor transfected PC3 cells and LNCaP cells in the G0-G1 phase was ∼23% (PC3) and 19% (LNCaP) less than that of NC inhibitor transfected cells, which paralleled with a ∼33% (PC3) and 25% (LNCaP) increase in the S phase (Fig. 2D, * *p*<0.05; ** *p*<0.01). These data indicate that let-7a inhibits PC3 proliferation by inducing cell-cycle arrest at G1/S phase.

### Let-7a targets 3′UTR of E2F2 and CCND2

**Figure 3:**
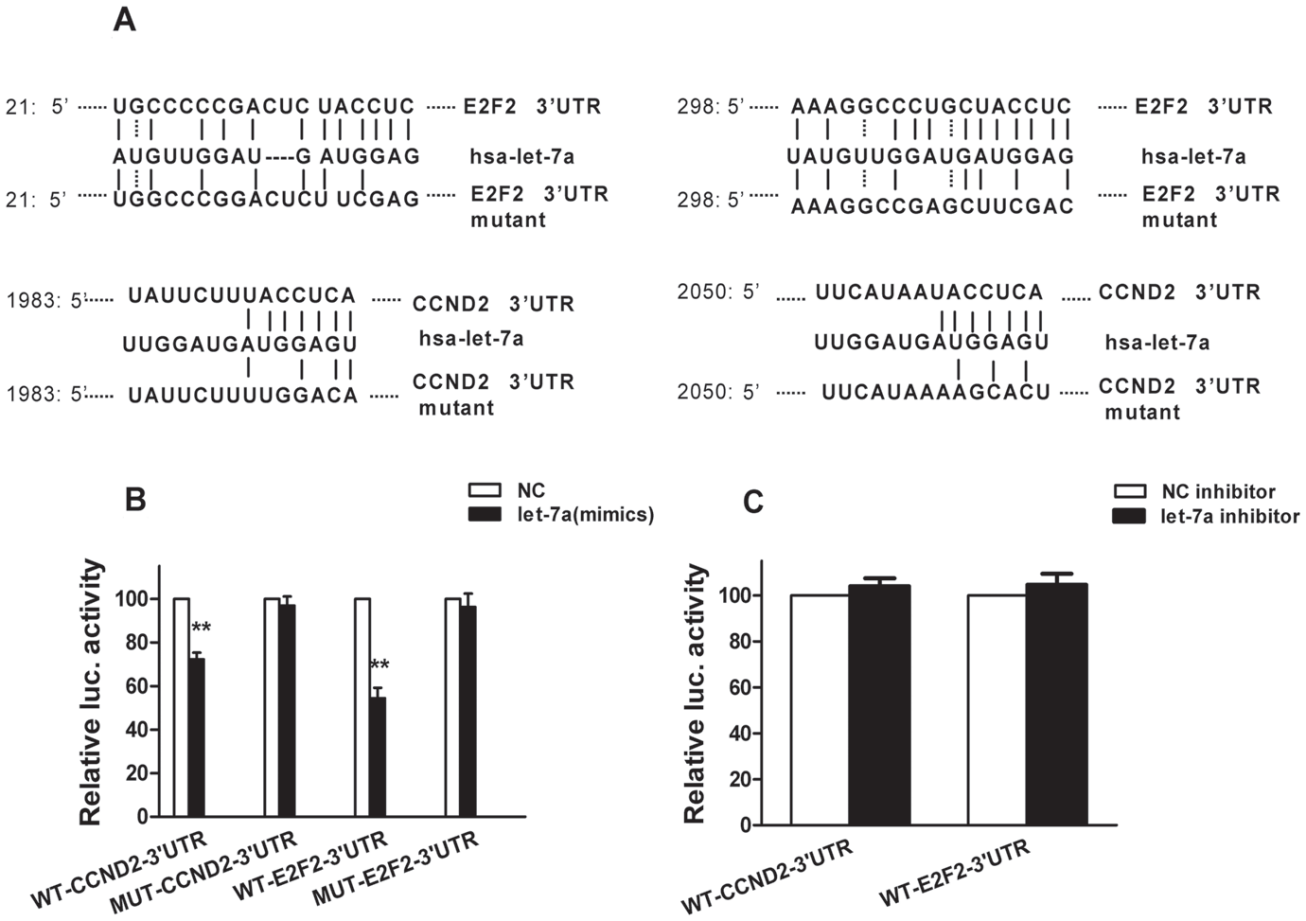
Luciferase activity using wild-type and mutated CCND2 and E2F2 3′UTR constructs. (**A**) Binding sites on E2F2 and CCND2 for let-7a with the corresponding mutated E2F2 and CCND2 sequences. (**B**) Relative luciferase activity of human embryonic kidney cells HEK293A after transfection with NC or let-7a (mimics) and then also co-transfected with pRL-TK control vector, or pGL3 expression vector containing either WT-CCND2-3′UTR or MUT-CCND2-3′UTR or WT-E2F2-3′UTR or MUT-E2F2-3′UTR. (**C**) Relative luciferase activity of human embryonic kidney cells HEK293A after transfection with NC inhibitor or let-7a inhibitor and also co-transfected with the pRL-TK control vector, or the pGL3 expression vector containing either WT-CCND2-3′UTR or WT-E2F2-3′UTR. Values represent means from three separate experiments and error bars represent the SEM. (** *p*<0.01; Mann-Whitney-*U* test).

Figure 3A shows the sequences of the 3′ UTRs of E2F2 and CCND2 that represent the binding sites for let-7a. The corresponding sequences of the mutated 3′ UTRs of E2F2 and CCND2 are shown as well. No reduction of luciferase activity was observed in HEK293A cells transfected with let-7a (mimics) and mutated CCND2 or E2F2. But greater than 30% reduction of luciferase activity was observed with wild-type CCND2 and ∼50% reduction of luciferase activity was observed with wild-type E2F2 (Fig. 3B, **p<0.01). Compared to NC inhibitor, there is no significant difference in luciferase activity when HEK293A cells were transfected with the let-7a inhibitor and wild-type CCND2 or E2F2 (Fig. 3C). These data support that E2F2 and CCND2 are direct targets of let-7a and endogenous let-7a in HEK293A has no interference to our experience.

### Let-7a inhibits expression of E2F2, CCND2

**Figure 4:**
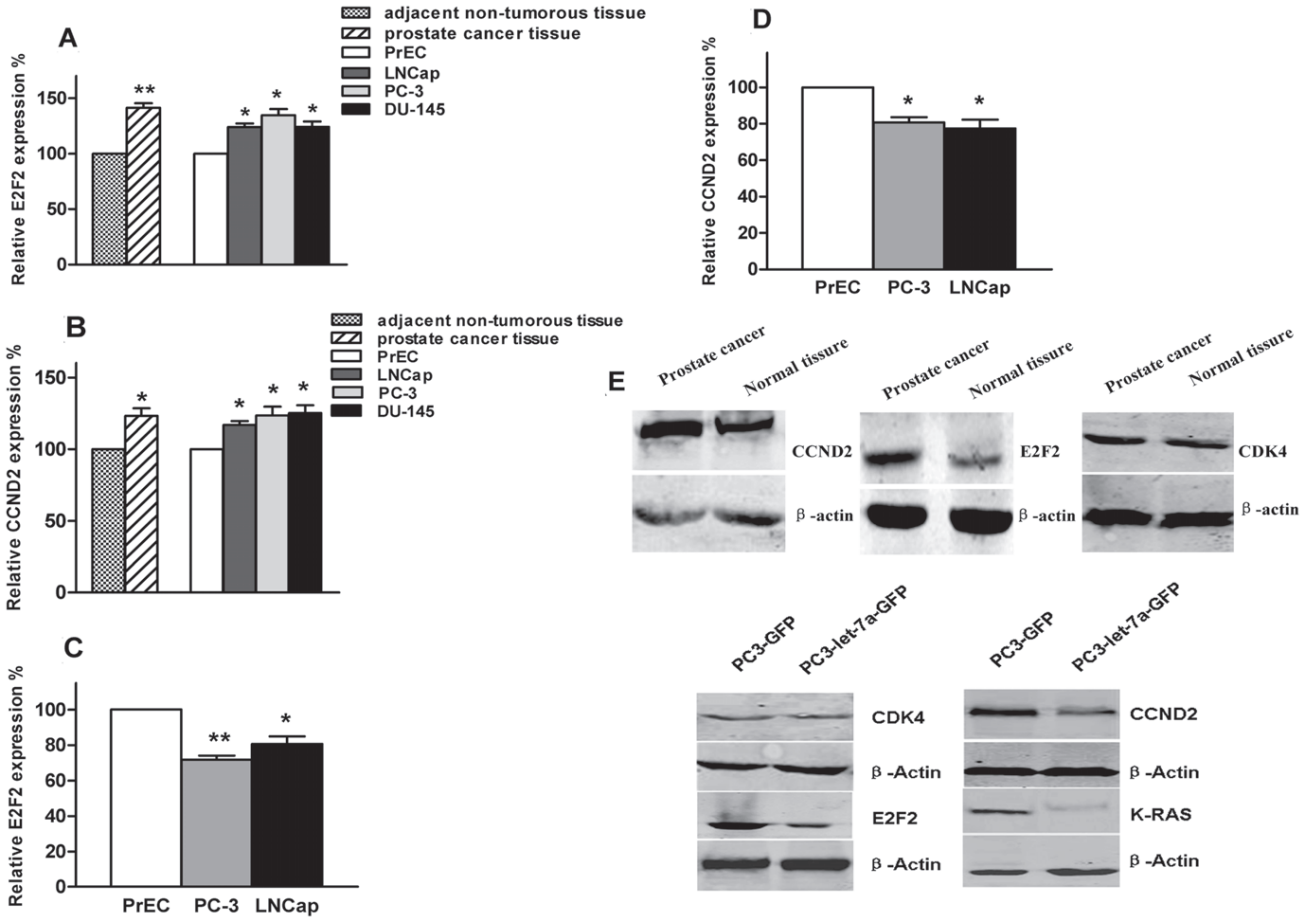
Effect of let-7a on expression of E2F2 and CCND2. (**A**) Real-time RT-PCR show that relative expression level of E2F2 in prostate cancer tissues and in LNCaP, PC3, and DU-145 cells is much higher than in their adjacent non-cancerous specimens and PrEC cells (* *p*<0.05, ** *p*<0.01; Mann-Whitney-*U* test). (**B**) Real-time RT-PCR show that relative expression level of CCND2 in prostate cancer tissues and LNCaP, PC3, and DU-145 cells is much higher than in their adjacent non-cancerous specimens and PrEC cells (* *p*<0.05; Mann-Whitney-*U* test). (**C**) Real-time RT-PCR show that relative expression level of E2F2 in LNCaP and PC3 cells is less than in PrEC cells after transfected with let-7a (* *p*<0.05, ** *p*<0.01; Mann-Whitney-*U* test). (**D**) Real-time RT-PCR show that relative expression level of CCND2 in LNCaP and PC3 cells is less than in PrEC cells after transfected with let-7a (* *p*<0.05; Mann-Whitney-*U* test). All the values represent means from three separate experiments and error bars represent the SEM. GAPDH was measured in parallel and used to normalize the expression levels of E2F2 and CCND2 in each experiment. (**E**) Western blotting show that no change of CDK4 expression between prostate cancer tissues and their adjacent non-cancerous specimens, or between PC3-let-7a-GFP cells and PC3-GFP cells. The expression levels of E2F2 and CCND2 is higher in prostate cancer tissues than in their adjacent non-cancerous specimens. After transfected with let-7a, the expression levels of E2F2, CCND2, and k-ras decreased dramatically in PC3-let-7a-GFP cells compared with that in PC3-GFP cells. β-actin was used the loading control.

Real-time RT-PCR shows that mRNA relative expression of E2F2 and CCND2 in prostate cancer tissues and in LNCaP, PC3, and DU-145 cells are up-regulated compared with their adjacent non-cancerous specimens and PrEC cells (Fig. 4A, B, * *p*<0.05; ** *p*<0.01). But compared to PrEC, mRNA expression of E2F2 and CCND2 are down-regulated after transfected with let-7a in PC3 and LNCaP cells (Fig. 4C, D, * *p*<0.05; ** *p*<0.01). Western blotting results showed no change of CDK4 protein expression between prostate cancer tissues and their adjacent non-tumorous tissues or between PC3-let-7a-GFP cells and PC3-GFP cells, the expression levels of E2F2, CCND2 increased in prostate cancer tissues compared with their adjacent non-tumorous tissues, but the expression levels of E2F2, CCND2, and k-ras decreased dramatically in PC3-let-7a-GFP cells compared with that in PC3-GFP cells (Fig. 4E). k-ras, a previously-defined molecular target of let-7a[7] was used as a positive control for these experiments. These data support that let-7a down-regulating the mRNA expression of E2F2 and CCND2 and repress protein translation of them.

### Let-7a inhibits tumor growth in nude mice xenograft model

**Figure 5:**
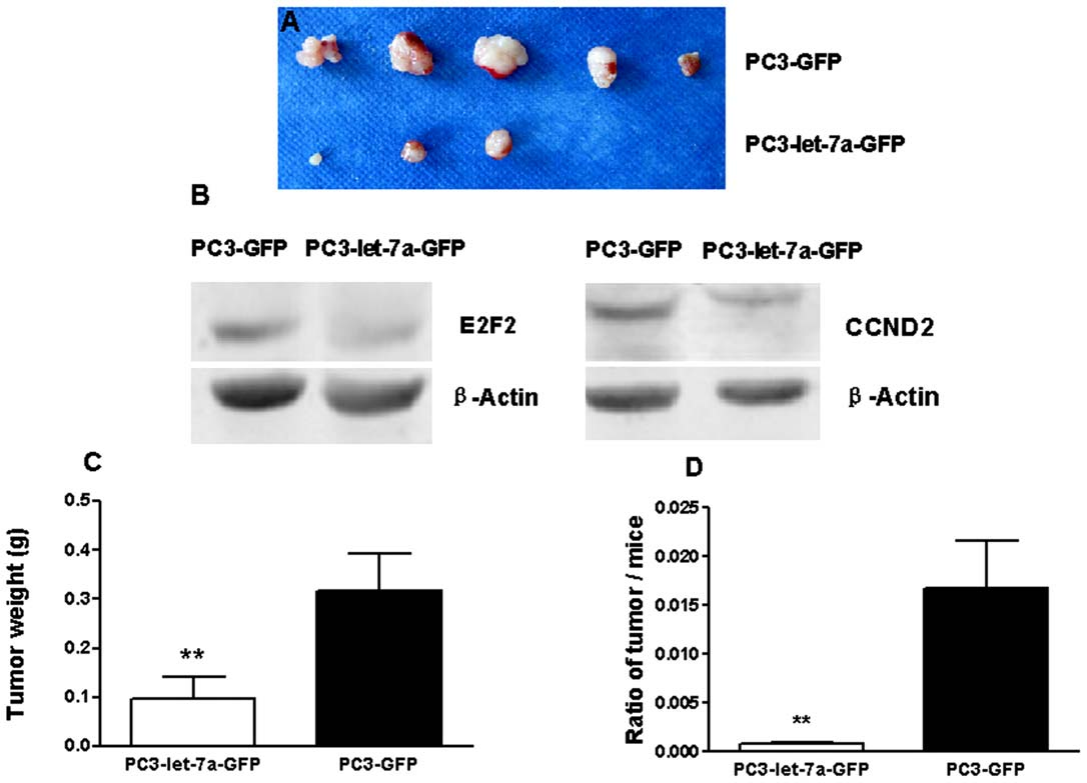
Effect of let-7a on tumorigenicity in xenograft models. (**A**) Photograph of excised tumors 4 weeks after implantation. The upper row shows tumors excised from mice injected with PC3-GFP cells. The lower are tumors excised from mice injected with PC3-let-7a-GFP cells. Of mice injected with PC3-let-7a-GFP cells, no tumor was detected in one mouse and one mouse died 2 days after injection. (**B**) Western blotting showed that the expression of E2F2 and CCND2 decreased dramatically in tumors excised from PC3-let-7a-GFP-tumor-bearin mice compared to those with PC3-GFP tumors. β-actin was used as an internal control. (**C**) Tumor weight and (**D**) ratio of tumor weight/body weight were determined when mice were sacrificed. Values represent means and error bars represent the SEM. (** *p*<0.01; Mann-Whitney-*U* test).

Nude mice bearing PC3-let-7a-GFP or PC3-GFP xenografts were sacrificed 4 weeks after innoculation. Tumors were excised and measured (Fig. 5A). Western blotting showed that expression levels of E2F2 and CCND2 decreased dramatically in PC3-let-7a-GFP tumors compared with PC3-GFP tumors (Fig. 5B). The weight of PC3-let-7a-GFP tumors was ∼80% lighter than PC3-GFP tumors (Fig. 5C, *p*<0.01). Furthermore, the ratio of tumor weight/body weight in mice bearing PC3-let-7a-GFP tumors was only ∼6% of the ratio from mice bearing PC3-GFP tumors (Fig. 5D, *p*<0.01), which provides strong evidence that let-7a can inhibit tumor growth *in vivo*.

## Discussion

The inability of a cell to regulate its growth and proliferation is a distinctive feature of cancer. As critical molecules in cell-cycle regulation, E2F proteins are downstream of growth-factor signaling cascades. They influence cell growth and proliferation through their ability to regulate genes involved in cell-cycle progression [10], [11]. E2F2 is a member of the E2F family of transcription factors, and has been well-characterized as a regulator of the G1-to-S-phase transition [12]. Previous reports indicate that E2F2 has strong oncogenic capacity and can promote cell-cycle progression. Cell lines transfected with E2F2 proliferate at twice the rate of control cells [13]. For proper progression through the cell cycle, phosphorylation activity of cyclin dependent kinase (Cdk) is essential. CCNDs are expressed in the early G1 phase and bind Cdk4 and Cdk6 [14]. The activation of these kinases results in phosphorylation of other critical cell-cycle regulators such as pRb. pRb then activates E2Fs and allows S-phase entry. Accordingly, aberrant expression of CCND2 and E2F2 will lead to abnormal cellular proliferation. Overexpression of CCND2 was reported in ovarian granulose cell tumors, gastric cancer and colon cancer [15]–[17]. Similarly, upregulation of E2F2 was found in astrocytomas of different grades [18].

miRNAs post-transcriptionally regulate gene expression by binding to the 3′UTR of target mRNAs. Binding leads to the degradation of target mRNAs and reduced translation of target proteins. miRNA activity also affects the expression of genes that are downstream of direct targets and can lead to changes in global protein expression profiles. Therefore, miRNAs potentially play a critical role in the progression of human cancer. A great deal of evidence supports that aberrant expression of miRNAs occurs in diverse types of human cancer, and at different stages of cancer progression [19], [20]. let-7 has been reported to act as a tumor suppressor in some cancer types, such as lung and colon cancer[9], [21]–[23] and that reduced expression levels of let-7 is correlated with poor clinical prognosis [24].

Although this correlation between let-7 expression and disease is strongest in lung tissue, where let-7 expression is high, our study finds that let-7 causes cell cycle defects in prostate cancer cell line as well. We found that let-7a expression level is down-regulated dramatically in prostate cancer tissue and cells lines. Protein and mRNA expression levels of E2F2 and CCND2 are down-regulated in PC3 and LNCaP cells after transfection with let-7a. Our xenograft models of prostate cancer also confirm our *in vitro* results and show that let-7a has the ability to inhibit prostate tumor development *in vivo*. Our dual-luciferase reporter assay verified that E2F2 and CCND2 are direct targets of let-7a. Also, other genes associated with cell-cycle regulation have been reported to be repressed directly or indirectly by let-7. These include CDC34, which promotes the degradation of cyclin dependent kinase (CDK) inhibitor 1B and CCNA2, which promotes G1-S and G2-M phase transitions [25]. Our founding expands the family of let-7 targets, and the identifying the molecular pathways affected in cancer could further reveal the mechanisms by which let-7a inhibits cell division. Though much is still to be learned about the role of let-7a in prostate cancer tumorigenesis, let-7a provides us a new way of prostate cancer treatment via its ability to inducing cell cycle arrest and inhibiting cell growth.

## Supporting Information

Table S1 Clinic pathological factors of 26 patients currently used.
(0.08 MB DOC)

Text S1 Sequences of synthetic Hsa-let-7a, negative control, Hsa-let-7a inhibitor and Inhibitor negative control.
(0.02 MB DOC)
