# Supplementary figures and images for: MicroRNA Let-7a Inhibits Proliferation of Human Prostate Cancer Cells In Vitro and In Vivo by Targeting E2F2 and CCND2
Source: PLoS One. 2010 Apr 14;5(4):e10147. doi: 10.1371/journal.pone.0010147 (PMC2854685; doi:10.1371/journal.pone.0010147)

Table 1. Clinic pathological factors of 26 patients currently used.


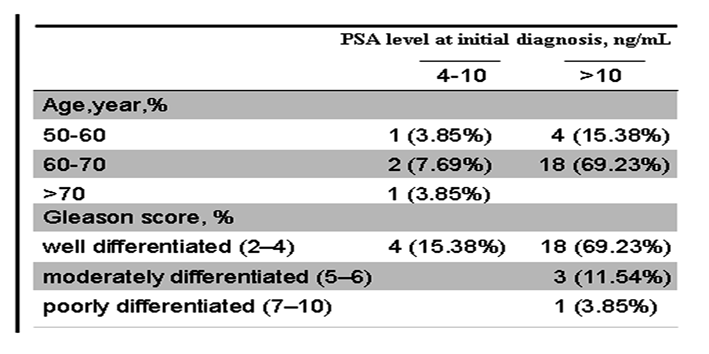

Supplement: Table S1 — Clinic pathological factors of 26 patients currently used. (0.08 MB DOC) [file pone.0010147.s001.doc]
