# Supplementary material for: MicroRNA Let-7a Inhibits Proliferation of Human Prostate Cancer Cells In Vitro and In Vivo by Targeting E2F2 and CCND2
Source: PLoS One. 2010 Apr 14;5(4):e10147. doi: 10.1371/journal.pone.0010147 (PMC2854685; doi:10.1371/journal.pone.0010147)
Supplement: Text S1 — Sequences of synthetic Hsa-let-7a, negative control, Hsa-let-7a inhibitor and Inhibitor negative control. (0.02 MB DOC) [file pone.0010147.s002.doc]

Sequences of synthetic Hsa-let-7a, negative control, Hsa-let-7a inhibitor and Inhibitor negative control:

Hsa-let-7a:

Sense:*5’-UGAGGUAGUAGGUUGUAUAGUU-3’*  Anti-sense:*5’-CUAUACAACCUACUACCUCAUU-3’*

Negative control:

Sense: *5’-UUCUCCGAACGUGUCACGUTT-3’*

Anti-sense: *5’-ACGUGACACGUUCGGAGAATT-3’*

Hsa-let-7a inhibitor:

*5’-AACUAUACAACCUACUACCUCA-3’*

Inhibitor negative control:

*5’-CAGUACUUUUGUGUAGUACAA-3’*
